# Association of serum concentrations of persistent organic pollutants (POPs) and risk of pre-eclampsia: a case–control study

**DOI:** 10.1186/s40201-016-0256-9

**Published:** 2016-11-24

**Authors:** Bita Eslami, Hossein Malekafzali, Noushin Rastkari, Batool Hossein Rashidi, Abolghasem Djazayeri, Kazem Naddafi

**Affiliations:** 1 Vali-e-Asr Reproductive Health Research Center, Tehran University of Medical Sciences, Tehran, Iran; 2 Center for Air Pollution Research (CAPR), Institute for Environmental Research (IER), Tehran University of Medical Sciences, 8th Floor, No. 1547, North Kargar Ave., Enghelab Square, Tehran, I.R. Iran; 3 Department of Community Nutrition, School of Nutritional Sciences and Dietetics, Tehran University of Medical Sciences, Tehran, Iran

**Keywords:** Persistent Organic pollutants (POPs), Polychlorinated Biphenyls (PCBs), Polybrominated Diphenyl Ethers (PBDEs), Pre-eclampsia, Case–control study

## Abstract

**Background:**

There is increasing evidence that persistent organic pollutants (POPs) may contribute to pre-eclampsia. The objective of this study was to evaluate the association between Polychlorinated Biphenyls (PCBs) and Polybrominated Diphenyl Ethers (PBDEs) as POPs with pre-eclampsia.

**Methods:**

This case–control study was performed in the three general university hospitals of Tehran University of Medical Sciences. Serum samples were collected from cases (*n* = 45) who had diagnosed with preeclampsia and from control samples (*n* = 70) with normal pregnancy and attended the same hospital for a routine prenatal visit at the third trimester of pregnancy. Pollutants levels were analyzed by Gas Chromatography Mass Spectrometry (GC/MS).

**Results:**

Mean participant age was 27.3 ± 5.39 with median 27. As the main independent variable, total POPs manifested with adjusted OR equal to 1.54 (95 % CI: 1.26–1.87, *p*-value <0.0001), which was significantly associated with pre-eclampsia. The adjusted OR proved a statistically significant association between total PCBs 1.77 (95 % CI: 1.34–2.32) and total PBDEs (OR = 2.19; 95 % CI: 1.39–3.45, *p*-value = 0.001) with pre-eclampsia considering confounding variables (maternal age, pre-pregnancy body mass index (BMI), gestational age, weight gain during pregnancy and total lipids in maternal serum). Finally, pre-pregnancy BMI and weight gain during pregnancy had a positive association with pre-eclampsia and gestational age yielded a negative association with pre-eclampsia in all analysis.

**Conclusion:**

Our data indicate the association between total POPs, total PBDEs, and total PCBs with pre-eclampsia, even after controlling for the effects of a number of potentially confounding factors. Further investigation about route of exposure and the trend of POPs especially in pregnant women is needed.

## Background

Persistent organic pollutants (POPs) are characterized by their ability to persist in the environment, low water and high lipid solubility, slow degradation and their bio-magnification in the food chain [1]. Polychlorinated Biphenyls (PCBs) and Polybrominated Diphenyl Ethers (PBDEs) are classified as POPs.

PCB have been widely used in heat exchange fluids, in electronic transformers and capacitors and used as an additive in paint, carbonless copy paper, sealants and plastics [2]. PBDEs are synthetic chemicals used as flame retardants in a variety of consumer products such as computer circuit boards and casings, television and radios, furniture, textiles, automobiles and construction materials [3]. The chemical structure and properties of PBDEs are similar to those of PCBs, which were banned in several countries [4].

The ability of POPs to co-distil, volatilize from landfills into the atmosphere and resist degradation, makes atmospheric transport of these chemicals as the primary mode of global distribution [5]. So people around the world are exposed to these chemicals.

There is growing evidence that POPs contribute to blood pressure and hypertension in general population [6–14]. One of the most common pregnancy problems leading to maternal and perinatal morbidity and mortality worldwide is hypertensive disorders, that increase perinatal mortality fivefold especially in developing countries [15]. Pre-eclampsia is one of the categories of hypertensive disorders which are classified by the Working Group on High Blood Pressure in Pregnancy [15]. Ten percent of pregnant women have high blood pressure and pre-eclampsia complicates 2 to 8 % of pregnancies [16].

The National High Blood Pressure Education Program of the American College of Obstetrics and Genecology defined pre-eclampsia as a pregnancy-specific syndrome usually occurs after 20 weeks’ gestation and determined by increased blood pressure (>140 mm Hg systolic or >90 mm Hg diastolic) accompanied by proteinuria [17]. Despite advances in perinatal care, frequency of pre-eclampsia has not changed and during past decade, research addressing this disorder has been increased [17].

Few studies investigated association of chemical pollutants and risk of hypertensive disorders during pregnancy [2, 18–22] and magnitude of such effects remains largely unknown. Evidence suggested defective vascular remodelling of the spiral arteries in the first half of pregnancy causes pre-eclampsia [23] and Dubey et al. study manifested the environmental oestrogens such as synthetic xenoestrogens that are widely used in industrial compounds like PCBs may induce abnormalities in vascular function and structure by mimicking or antagonizing the vascular effects of oestardiol, leading to pre-eclampsia [24]. Recent study also has been reviewed dioxins and related pollutants effects on the placental angiogenesis and vascular remodelling during embryo and foetal development by reducing blood flow and circulatory function, that possibly leading to pre-eclampsia [25]. Since this problem is sever complication for mother and infant and several studies have suggested that women who develop pre-eclampsia are at increased risk of cardiovascular disease later in life [26, 27], more evaluation of this complication is necessary.

For research about pollutants in pregnant women it should be noted, because of different trends of POPs in each trimester of pregnancy [28, 29], the investigation of POPs within a pregnancy should be limited to one trimester for a more accurate evaluation. Meanwhile, previous studies reported that breastfeeding is one of the main routes for pollutants excretion [30, 31] and breastfeeding has a significant effect on decreasing the levels of organochlorine pesticides and PCBs in serum [32]. Therefore, measurement of POPs in primiparous women gives us better estimation of pollutants in human samples [33, 34].

On the other hand, pre-eclampsia as a heterogenous disorder has different pathogenesis especially in nulliparous women [17]. One study published a model estimating a heritability of 31 % for pre-eclampsia [35]. However, studies that investigated the relationship between chemical pollutants and pre-eclampsia did not adjust family history of pre-eclampsia in first degree relatives as an important component. Meanwhile they did not consider parity and history of breast feeding in pregnant women.

Therefore the objective of the present study was to find the association of POPs (PCBs and PBDEs) with risk of pre-eclampsia in a case–control study in primiparous women who had not reported family history of pre-eclampsia in first degree relatives.

## Methods

### Study sample

This case control study was approved by Institutional review board of Tehran University of Medical Sciences and all subjects signed their informed consent to participate in the study. The study population consisted of pregnant women enrolled in the three general university hospitals (Arash, Vali-e-Asr, MirzaKouchak Khan) in different districts of Tehran, Iran during a prenatal visit. These hospitals were chosen in order to collect samples from almost all districts of city.

All cases (*n* = 45) were selected from pregnant women who had been diagnosed with pre-eclampsia during the routine prenatal care. Pre-eclampsia was defined at least two blood pressure measurement that were ≥ 90 mm Hg diastolic or ≥ 140 mm Hg systolic within 14 days of one another after 20 weeks of gestation accompanied by proteinuria at any level. They had not reported previous abnormal blood pressure. Pre-eclampsia was diagnosed and confirmed by at least two specialist physicians. Blood pressure in all participants was measured twice using sphygmomanometers in either right or left arms after the woman was seated and at rest for a minimum of 15 min by an appropriate cuff size.

Controls (*n* = 70) consisted of pregnant women attending the same hospitals for a routine prenatal visit in pregnancy with completely normal test results.

All cases and controls were primiparous mothers with singleton baby at the third trimester of pregnancy. Participants had not reported any of the following criteria: previous family history of pre-eclampsia in first degree relatives (mother, sister or daughter), family history or self-history of diabetes, gynecology and obstetrics problems, previous history of metabolic and endocrine disorders, renal failure, liver and thyroid disease, chronic autoimmune disease, and anti-phospholipid syndrome.

### Collection of serum samples and data

Sample collection took place from September 2013 until August 2015. Hospital nurses collected 10 ml blood samples into two 5-mL vacuum blood collection polypropylene tubes from the cubital vein. The serum samples were separated by centrifugation at 3000 g for 15 min within 4 h and transferred to new blood tubes into two parts. A part of serum sample was used to measure the total cholesterol and triglyceride and the remaining parts were stored for measuring POPs. The researcher transferred all collected samples from different hospitals to the Vali-e-Asr hospital laboratory of the Tehran University of Medical Sciences. All serum samples were coded with numbers and then transferred to the laboratories for measurement of triglycerides and cholesterol or POPs levels and laboratory personals were blind about subject group and only the main investigator knows about coding numbers. The samples were iced-packed and stored at −70 ° C to avoid thawing. A staff member of the Vali-e-Asr hospital laboratory then analyzed the serum samples for total cholesterol and triglyceride levels using enzymatic kits (Pars Azmoon, Iran).

The trained questioner in each hospital recorded individual information about maternal age, pre-pregnancy weight, height, residual status, gestational age, weight gain during pregnancy, supplement consumption at pregnancy, occupation, smoking and alcohol consumption, physical activity and walking by face- to- face interview. Physical activity was defined as having more than 10 min of any activity per day accompanied by increased heartbeat and breathing. Walking was expressed as having at least 10 min of walking per day and total day per week was calculated. Pre-pregnancy body mass index (BMI) was calculated as weight (before pregnancy) in kilograms divided by height in meters squared.

### Serum extraction and instrument

Serum samples were thawed on ice overnight before preparation. The analytical procedure details for PBDEs and PCBs were described elsewhere [36]. Briefly, the samples were fortified with Hexachlorobenzene (internal standard) and then mixed with 0.5 ml of pure formic acid and ultrasonicated for 10 min. Serum and formic acid mixture was applied to a combined SPE cartridges comprised of poly divinyl benzene-co-N-vinyl pyrrolidone and Phenomenex Strata-X sorbent (50:50 w/w 200 mg) and filtered. The eluent was cleaned up by a serially combined SPE cartridges including Sep-pak® Light Silica cartridges and Sep-Pak® plus Florisil which were placed underneath the SPE cartridges, and after SPE conditioning steps the analytes were eluted with dichloromethane under vacuum. The residue was reconstituted in isooctane and analyzed by Gas Chromatography Mass Spectrometry (GC/MS).

PCB and PBDE congeners were analyzed using an Agilant 6890–5973 GC-MS (Agilant Technologies, Palo Alto, CA, USA). Split/splitless injector set at 250 °C. GC separation was performed by a HP-5MS column, (30× 0.25 mm i.d 0.25 μm film thickness). The GC oven temperature was started at 90 °C and held at that temperature for 1 min. Temperature was then increased to 150 °C at a rate of 50 °C/min and held for 1 min. Finally, oven temperature was increased to 330 °C at a rate of 8 °C/min and held for 3 min. The flow rate of carrier gas, helium, was set at 1.0 ml/min. Ion source and analyzer temperatures were set at 250 and 150 °C, respectively. Transfer line temperature was set at 280 °C.

### Identification and quantification of PCBs and PBDEs

Laboratory assays of POPs were performed at the accredited Faroogh laboratory. In this study, we focused on the ten most abundant PCB congeners (International Union of Pure and Applied Chemistry (IUPAC) congeners 28, 52, 74, 99, 101, 118, 138, 153, 180 and 187) as well as eight PBDE congeners (IUPAC congeners 28, 47, 99, 100, 153, 154, 183 and 209). PCB and PBDE standard solution that contained our study congeners with more than 95 % purity were purchased from Accustandard (New Haven, CT, USA). The limit of detection (LOD) for each PCB congener was 0.05 ng/L and the LOD of each PBDE congeners was 0.1 ng/L. The limit of quantification (LOQ) for each PCB congener was 0.1 ng/L and the LOQ of each PBDE congeners was 0.2 ng/L. The concentration of some congeners in serum samples were less than LOD. We substituted these samples with congener specific LOD divided by the square root of two before analyses. Because POPs are highly lipophilic and predominantly carried by blood lipids, in order to lipid-standardized PCB and PBDE concentrations, the lipid content in serum samples estimated from the total cholesterol and triglycerides concentration using Philips formula [37]. Therefore, the serum concentration of PCBs and PBDEs were expressed as nanorgrams per gram of plasma total lipids.

### Statistical analyses

Statistical analyses were performed using the SPSS software (SPSS, Version 16, SPSS, Inc., IL, USA). We tested differences between the means by Student’s *t*-test or Mann–Whitney *U* test, when appropriate. Categorical variable differences were tested by Chi-square (*χ* ^2^) test or Fisher’s exact test. The significance level was set at *p*-value less than 0.05, and all tests were two-sided. Total PCBs and total PBDEs were calculated by summing concentrations of their congeners. Total POPs was computed by summing concentrations of total PCBs and PBDEs.

All contaminant concentrations were continues variables for analyses. Multivariate binary logistic regression was used to estimate Odds ratio (OR) and 95 % confidence interval (CI) for the associations between PCBs, PBDEs and total POPs with pre-eclampsia. Potential confounders including maternal age (yrs), pre-pregnancy BMI (kg/m^2^), gestational age (wks), weight gain during pregnancy (kg), and total lipids in maternal serum (mg/dl) were adjusted. Variables were selected a priori for inclusion in multivariate models on the basis of the association with pre-eclampsia in univariate analyses (*p*-value < 0.20) or on the basis of evidence of an association from the literature (total lipid).

## Results

The response rate in normal group was 83.4 % (70/82) and in pre-eclampsia group was 93.8 % (45/48). The mean participants ‘age in this study was 27.3 ± 5.39 with median 27. Table 1 summarizes the characteristics of the study population. As we expected pre-pregnancy BMI was statistically significant higher in pre-eclampsia group (26.2 ± 4.86 vs 22.9 ± 4.17, *p*-value <0.001). None of the participants reported any physical activity during pregnancy. Previous alcohol consumption was reported only by one woman. No cases of smoker were observed in both groups. Therefore, we reported only the number of passive smokers in the Table 1.

**Table 1:** Baseline characteristics of case (pre-eclampsia) and control groups

Variables	Pre-eclampsia	Control	*P*-value
	(*n* = 45)	(*n* = 70)	
Age (yrs)	28.2 ± 6.27	26.7 ± 4.69	0.14
Gestational age (wks)	33.6 ± 3.50	35.1 ± 3.09	**0.01**
Weight gain (kg)	14.4 ± 5.60	13 ± 5.48	0.17
Pre-pregnancy BMI (kg/m2)	26.2 ± 4.86	22.9 ± 4.17	**<0.001**
Triglyceride (mg/dl)	270 ± 101.14	263.5 ± 100.31	0.74
Total cholesterol (mg/dl)	212.7 ± 41.53	222.4 ± 48.22	0.27
Total Lipids (mg/dl)	815.2 ± 161.93	830.7 ± 174.44	0.63
Education			0.76
Master	1 (2.2)	0 (0)	
Bachelor	10 (22.2)	13 (18.6)	
Associate	2 (4.4)	3 (4.3)	
Diploma	21 (46.7)	36 (51.4)	
Under diploma	11 (24.4)	18 (25.7)	
Passive smoker	14 (31.1)	15 (21.4)	0.24
Alcohol consumption	0 (0)	1 (1.4)	1
Walking (day/wk)	3.2 ± 2.84	3.5 ± 2.90	0.52
Job			0.42
Housewife	39 (86.7)	64 (91.4)	
Others	6 (13.3)	6 (8.6)	
Use of supplement			
Routine supplementation	11 (24.4)	10 (14.3)	0.17
Routine plus Calcium	21 (46.7)	34 (48.6)	0.84
Routine plus Multivitamin	25 (55.6)	47 (67.1)	0.21
Routine plus Omega 3	4 (8.9)	11 (15.4)	0.29

Date shown as mean ± SD and numbers with percentages in parenthesis

*P*-value refers to *t*-test, chi-square test and Fisher exact test when appropriate. All significant *P*-value was bolded

All pregnant women in this study received routine iron and folic acid supplementation during their pregnancy. In addition to routine supplementation, some of the participants also received other supplements during pregnancy such as calcium, multivitamin, or Omega-3. As indicated in the Table 1, there was not any significant difference in supplementation consumption between either of the groups (*p*-value > 0.05).

The arithmetic mean and detection rate of each congener was reported in Table 2. PCB 118, 138, 153 and 180 were detected in more than 90 % of serum samples. Meanwhile, the detection rate of PBDE 47, 99 and 153 were more than 80 %. PCB 74 was not detected in any samples. So it was deleted for further analysis. The distribution of total PCBs, total PBDEs and total POPs were not normal (Kolmogrove - Smirnove test, *p*-value < 0.05) and right-skewed. Table 3 manifested the arithmetic mean for the total PCBs (PCB with nine congeners) and total PBDEs (PBDE with eight congeners) were statistically significant higher in pre-eclampsia group in comparison to control (Mann- Whitney *U*-test; *p*-value < 0.001).

**Table 2:** Arithmetic means of PCB and PBDE congeners (ng/g lipid) and detection rate of congeners in each group

Congeners	Pre-eclampsia(*n* = 45)		Control (*n* = 70)		*P*-value*
	Detection rate	Mean ± SD	Detection rate	Mean ± SD	
**PCB28**	33 (73.33)	0.03 ± 0.02	40 (57.14)	0.01 ± 0.01	**<0.001**
**PCB52**	17 (37.78)	0.014 ± 0.014	18 (25.71)	0.010 ± 0.014	0.37
**PCB99**	26 (57.78)	0.02 ± 0.01	29 (41.43)	0.01 ± 0.01	0.06
**PCB101**	18 (40)	0.01 ± 0.01	14 (20)	0.008 ± 0.007	0.05
**PCB118**	42 (93.33)	0.04 ± 0.02	51 (72.86)	0.02 ± 0.01	**<0.001**
**PCB138**	45 (100)	1.51 ± 1.03	70 (100)	0.81 ± 0.66	**<0.001**
**PCB153**	45 (100)	1.84 ± 1.25	70 (100)	1.13 ± 1.02	**<0.001**
**PCB180**	44 (97.78)	0.19 ± 0.17	45 (64.29)	0.05 ± 0.05	**<0.001**
**PCB187**	38 (84.44)	0.07 ± 0.07	24 (34.29)	0.02 ± 0.03	**<0.001**
**PBDE28**	35 (77.78)	0.05 ± 0.06	14 (20)	0.01 ± 0.01	**<0.001**
**PBDE47**	38 (84.44)	0.10 ± 0.12	36 (51.43)	0.05 ± 0.07	**0.001**
**PBDE99**	39 (86.67)	0.06 ± 0.07	25 (35.71)	0.02 ± 0.03	**<0.001**
**PBDE100**	10 (22.22)	0.01 ± 0.008	8 (11.43)	0.01 ± 0.004	0.71
**PBDE153**	45 (100)	1.88 ± 1.52	70 (100)	1.07 ± 0.83	**0.001**
**PBDE154**	6 (13.33)	0.01 ± 0.007	4 (0.54)	0.01 ± 0.003	0.35
**PBDE183**	24 (53.33)	0.03 ± 0.03	24 (34.29)	0.02 ± 0.02	0.13
**PBDE209**	7 (15.56)	0.01 ± 0.006	14 (20)	0.01 ± 0.007	0.86

Date shown as mean ± SD and numbers with percentages in parenthesis

*P-value refers to comparison of means by Mann–Whitney *U*-test between congener’s concentrations. Significant *P*-value was bolded

**Table 3:** Comparison between total PCBs, PBDEs and POPs (ng/g lipid)

	Pre-eclampsia		Control		*P*-value
Variables	(*n* = 45)		(*n* = 70)		
	Mean ± SD	Median	Mean ± SD	Median	
Total PCBs	3.72 ± 2.37	2.86	2.07 ± 1.66	1.50	**<0.001**
Total PBDEs	2.15 ± 1.64	1.51	1.20 ± 0.90	0.95	**<0.001**
Total POPs	5.87 ± 3.54	5.15	3.28 ± 2.23	2.70	**<0.001**

Significant *P*-value was bolded

We assessed the possibility of correlation between congeners. Spearman correlations between PCB congeners shows PCB180, 187 (correlation coefficient: *r* = 0.74) and PCB 138, 153 (*r* = 0.80) and PCB 28, 118 (*r* = 0.76) were strongly correlated with each other (all *p*-value <0.001). Among PBDE congeners and across pollutants classes, highly correlated congeners were not observed (all correlation coefficient was less than 0.70).

The collinearity for all PCBs and PBDEs congeners that were checked using variation inflammation factor (VIF) manifested all VIF less than 5.

Table 4 shows, OR and 95 % CI of pollutants and other variables in relation to pre-eclampsia with using logistic regression analysis. Total POPs, total PCBs, and total PBDEs entered the model separately (Model 1 to 3). As the main independent variable, total POPs manifested with OR equal to 1.54 (95 % CI: 1.26-1.87, *p*-value < 0.001), which was significantly associated with pre-eclampsia. The adjusted OR proved a statistically significant association between total PCBs 1.77(95 % CI: 1.34–2.32) and total PBDEs (OR = 2.19; 95 % CI: 1.39–3.45, *p*-value = 0.001) with pre-eclampsia, too. When we performed logistic regression analysis with total PCBs and total PBDEs simultaneously (Model 4), the adjusted OR of total PCBs did not change. However, total PBDEs have no statistically significant effect on pre-eclampsia (OR = 1.52; 95 % CI: 0.90–2.58, *p*-value = 0.12).

**Table 4:** Maternal serum concentrations of total POPs, total PCBs, and total PBDEs in relation to odds of preeclampsia in four models

	Crude OR (95 % CI)	*P*-value	Adjusted OR (95 % CI)	*P*-value
Model 1				
Total POPs	1.40 (1.18–1.65)	**<0.001**	1.54 (1.26–1.87)	**<0.001**
Gestational age	0.87 (0.77–0.97)	**0.02**	0.74 (0.62–0.89)	**0.001**
Pre-pregnancy BMI	1.19 (1.08–1.32)	**0.001**	1.29 (1.14–1.46)	**<0.001**
Weight gain	1.05 (0.98–1.12)	0.17	1.11 (1.11–1.22)	**0.04**
Model 2				
Total PCBs	1.54 (1.22–1.95)	**<0.001**	1.77 (1.34–2.32)	**<0.001**
Gestational age			0.74 (0.62–0.88)	**0.001**
Pre-pregnancy BMI			1.28 (1.13–1.44)	**<0.001**
Weight gain			1.12 (1.02–1.23)	**0.01**
Model 3				
Total PBDEs	1.89 (1.30–2.75)	**0.001**	2.19 (1.39–3.45)	**0.001**
Gestational age			0.78 (0.67–0.92)	**0.003**
Pre-pregnancy BMI			1.25 (1.12–1.40)	**<0.001**
Weight gain			1.09 (0.99–1.19)	0.07
Model 4				
Total PCBs	1.35 (1.05–1.74)	**0.02**	1.77 (1.34–2.32)	**<0.001**
Total PBDEs	1.49 (0.97–2.30)	0.07	1.52 (0.90–2.58)	0.12
Gestational age			074 (0.62–0.88)	**0.001**
Pre-pregnancy BMI			1.28 (1.13–1.44)	**<0.001**
Weight gain			1.12 (1.02–1.23)	**0.01**

Model 1: Total POPs as an independent variable and confounding factors entered to the model

Model 2: Total PCBs as an independent variable and confounding factors entered to the model

Model 3: Total PBDEs as an independent variable and confounding factors entered to the model

Model 4: Total PCBs and total PBDEs as independent variables entered to the model simultaneously with confounding factors

Significant *P*-value was bolded

Pre-pregnancy BMI and weight gain during pregnancy had a positive association with pre-eclampsia and gestational age yielded a negative association with pre-eclampsia in all analysis (Table 4).

## Discussion

In the present study we reported the positive association between total POPs, total PBDEs, and total PCBs and pre-eclampsia in primiparous women who had no previous history of pre-eclampsia in first degree relatives. We found a positive association between pre-eclampsia and total PCBs. Interestingly; we also discovered a stronger association between serum concentrations of total PBDEs with pre-eclampsia, suggesting different or additional routes of exposure. The association remained significant, even after controlling confounding factors.

Furthermore, when considering the interaction between these two chemicals, the effect of PCBs was similar to previous analysis which was entered to the model separately. However the effect of PBDEs has been decreased and PCBs may block the toxic actions of total PBDEs (Table 4). On the other hand comparing the low risk of total POPs on pre-eclampsia with separate ORs of each pollutant, it seems that the two chemicals action on the pre-eclampsia does not occur via the same mechanism. Therefore, exposure to PCBs and PBDEs does not have an additive effect on the risk of pre-eclampsia.

In contrast to our study findings, previous studies have had different conclusions. A recent study by Savitz et al. that was more similar to our study revealed no association between serum level of total PCBs and risk of pre-eclampsia [19]. A comparison of our results with this study elicits differences in a variety of factors. First of all, they did not consider the participants’ family history of pre-eclampsia as a confounding variable. Secondly, they included pregnant women regardless of parity and history of breastfeeding as one of the main routes for pollutants excretion. Finally, the aforementioned research conducted analysis with different sample size and using categorical data instead of continues data.

Another study by Saunders et al. had results that conflicted with the present study. The authors reported no evidence of association between preeclampsia and Chlordecone as an organochlorine insecticide that has endocrine activities among pregnant women chronically exposed to this chemical [2].

The association between another category of pollutants (organophosphorous pesticide) and gestational hypertension not specifically pre-eclampsia, was reported in Italian population, which is consistent with our study findings [21]. Another study results, indicated that women living in households using biomass and solid fuels have higher likelihood of reporting pre-eclampsia, even after controlling for the effects of a number potentially confounding factors [20]. The result of this study supported the hypothesis of indoor pollutants effects on the risk of pre-eclampsia rather than outdoor pollutants. However, some studies reported the air pollution as an outdoor pollutant during pregnancy increase the risk of hypertensive disorders of pregnancy and pre-eclampsia [18, 22, 38].

In the present study we found the strong positive association between PBDEs and pre-eclampsia after adjustment of confounders. Since the PBDEs have been extensively used in indoor applications, indoor air concentrations of PBDEs are generally higher than outdoor levels and are considered as significant pollutants of the indoor environment [39]. On the other hand, the present study population consisted mostly of housewives without occupational exposure to POPs due to spending most of their time indoors. As a result, it seems that the most common route of exposure in our population is indoor pollutants such as air and dust.

As the capital city of Iran, Tehran is a highly polluted area. As we mentioned in previous studies pregnancy exposure to air pollution maybe associated with an increased risk of subsequent hypertensive disorders [18, 22, 38]. Since our target population (cases and controls) were living in Tehran, and distribution of both groups were consistent in different regions of the capital city, the exposure of both cases and controls to air pollution was fairly similar. Future studies that incorporate simultaneous measurements of indoor and outdoor pollutants are necessary to evaluate whether outdoor pollution is the main cause of pre-eclampsia in this population.

This study was conducted at the third trimester of pregnancy. Because of long half-life of these chemicals (2–10 year) [40], single measurement at the third trimester of pregnancy is reasonably representative of exposure throughout the pregnancy.

This study had some advantages. We collected samples from primiparous women to eliminate the effect of parity and breastfeeding as confounding factors. Meanwhile, in order to reduce the effects of genetics and better estimate the environmental impacts, we included women without family history of pre-eclampsia in first degree relatives. Because we may have missed the existence of genetic backgrounds in other relatives, a further evaluation of POPs with genetic assessments at the same time is necessary in future studies. Since, some studies reported the effect of the calcium supplementation with or without co-supplements and iron and folic acid consumption on reduction of the risk of preeclampsia [41, 42], we considered to supplementation intake during pregnancy in our participants and we did not find any significant differences in supplementation intake between two groups.

We should consider, the level of POPs are adjusted in total lipids in the present study and during pregnancy total cholesterol will increase on average 58 % and triglyceride will increase on average 145 % throughout the second and third trimester [43]. On the other hand, during pregnancy there is a physiological increase in plasma volume [44] and this could lead to a dilution of pollutants in the plasma of pregnant women. Therefore, after delivery and during lactation higher concentrations of pollutants are expected and this leads to higher exposure of the baby through breastfeeding. Further study is needed to compare the pollutants concentrations during and after pregnancy.

This study had some limitation: Firstly, we couldn’t collect enough preeclamptic samples during the study time due to strict inclusion criteria and impossibility of sampling in severe cases. Because, if there is severe pre-eclampsia, pregnancy should be terminated as soon as possible to save the mother’s life. Secondly, we have no information about anemia in this population as a major risk for pre-eclampsia [45]. The third limitation of our study was due to correlation of POPs with each other and it is not possible to determine the independent effect of each POPs on pre-eclampsia and different chemicals can modify the effects of each other on the risk of disease. Therefore, further research is needed to corroborate these findings and shed light on the biological mechanisms by which these types of chemicals may influence the pre-eclampsia.

## Conclusion

Our data indicate the association between total POPs, total PBDEs, and total PCBs, and pre-eclampsia even after controlling for the effects of a number of potentially confounding factors. Further investigation about route of exposure and the trend of POPs especially in pregnant women due to the effect on their infants and children’s health with more sample size is needed.

Even if this magnitude of association will be small, we can find the susceptible women for prevention of pre-eclampsia and prevention of cardiovascular disease later in life as a major global priority.

## Abbreviations

BMI: Body mass index
CI: Confidence interval
GC/MS: Gas Chromatography/Mass Spectrometry
IUPAC: International Union of Pure and Applied Chemistry
LOD: Limit of detection
OR: Odds ratio
PBDEs: Polybrominated diphenyl ethers
PCBs: Polychlorinated biphenyls
POPs: Persistent organic pollutants

## References

[1] Lee DH, Lee IK, Song K, Steffes M, Toscano W, Baker BA (2006). A strong dose–response relation between serum concentrations of persistent organic pollutants and diabetes: results from the National Health and Examination Survey 1999–2002. Diabetes Care.

[2] Saunders L, Kadhel P, Costet N, Rouget F, Monfort C, Thomé J-P (2014). Hypertensive disorders of pregnancy and gestational diabetes mellitus among French Caribbean women chronically exposed to chlordecone. Environ Int.

[3] Alaee M, Arias P, Sjödin A, Bergman Å (2003). An overview of commercially used brominated flame retardants, their applications, their use patterns in different countries/regions and possible modes of release. Environ Int.

[4] Ni K, Lu Y, Wang T, Kannan K, Gosens J, Xu L (2013). A review of human exposure to polybrominated diphenyl ethers (PBDEs) in China. Int J Hyg Environ Health.

[5] World health Organization. International programme on chemical safety. Polychlorinated biphenyls and terphenyls, 2nd ed. Geneva: World Health Organization; 1993.

[6] Yorita Christensen KL, White P (2011). A methodological approach to assessing the health impact of environmental chemical mixtures: PCBs and hypertension in the National Health and Nutrition Examination Survey. Int J Environ Res Public Health.

[7] Wu RS, Chan AK, Richardson BJ, Au DW, Fang JK, Lam PK (2008). Measuring and monitoring persistent organic pollutants in the context of risk assessment. Mar Pollut Bull.

[8] Peters JL, Fabian MP, Levy JI (2014). Combined impact of lead, cadmium, polychlorinated biphenyls and non-chemical risk factors on blood pressure in NHANES. Environ Res.

[9] Goncharov A, Pavuk M, Foushee HR, Carpenter DO (2011). Anniston Environmental Health Reseach C, Blood pressure in relation to concentrations of PCB congeners and chlorinated pesticides. Environ Health Perspect.

[10] Goncharov A, Bloom M, Pavuk M, Birman I, Carpenter DO (2010). Blood pressure and hypertension in relation to levels of serum polychlorinated biphenyls in residents of Anniston, Alabama. J Hypertens.

[11] Ha MH, Lee DH, Son HK, Park SK, Jacobs DR (2009). Association between serum concentrations of persistent organic pollutants and prevalence of newly diagnosed hypertension: results from the National Health and Nutrition Examination Survey 1999–2002. J Hum Hypertens.

[12] Huang X, Lessner L, Carpenter DO (2006). Exposure to persistent organic pollutants and hypertensive disease. Environ Res.

[13] Arrebola JP, Fernández MF, Martin-Olmedo P, Bonde JP, Martín-Rodriguez JL, Expósito J (2015). Historical exposure to persistent organic pollutants and risk of incident hypertension. Environ Res.

[14] Everett CJ, Frithsen I, Player M (2011). Relationship of polychlorinated biphenyls with type 2 diabetes and hypertension. J Environ Monit.

[15] Roberts JM, Pearson GD, Cutler JA, Lindheimer MD (2003). Summary of the NHLBI working group on research on hypertension during pregnancy. Hypertens Pregnancy.

[16] Duley L (2009). The global impact of pre-eclampsia and eclampsia. Semin Perinatol.

[17] Maryland B (2000). Report of the National blood Pressure Education Program Working Group on High Blood Pressure in Pregnancy. Am J Obstet Gynecol.

[18] Lee PC, Roberts JM, Catov JM, Talbott EO, Ritz B (2013). First trimester exposure to ambient air pollution, pregnancy complications and adverse birth outcomes in Allegheny County, PA. Matern Child Health J.

[19] Savitz DA, Klebanoff MA, Wellenius GA, Jensen ET, Longnecker MP (2014). Persistent organochlorines and hypertensive disorders of pregnancy. Environ Res.

[20] Agrawal S, Yamamoto S (2015). Effect of Indoor air pollution from biomass and solid fuel combustion on symptoms of preeclampsia/eclampsia in Indian women. Indoor Air.

[21] Ledda C, Fiore M, Santarelli L, Bracci M, Mascali G, D’Agati MG, et al. Gestational Hypertension and Organophosphorus Pesticide Exposure: A Cross-Sectional Study. BioMed Res Int. 2015;doi: 10.1155/2015/280891. Epub 3 Aug 2015. 10.1155/2015/280891 PMC4538315 26339602

[22] Wu J, Ren C, Delfino RJ, Chung J, Wilhelm M, Ritz B (2009). Association between local traffic-generated air pollution and preeclampsia and preterm delivery in the south coast air basin of California. Environ Health Perspect.

[23] Staff AC, Dechend R, Redman CW (2013). Review: Preeclampsia, acute atherosis of the spiral artries and future cardivascular disease:two new hypothesis. Placenta.

[24] Dubey KR, Rosselli M, Inthurn B, Keller PJ, Jackson EK (2000). Vascular effects of environmental oestrogens: implications for reproductive and vascular health. Hum Reprod Update.

[25] Wu Y, Chen X, Zhou Q, He Q, Kang J, Zheng J, Wang K, Duan T (2014). ITE and TCDD differentially regulate the vascular remodeling of rat placenta via the activation of AhR. PLoS One.

[26] Lind JM, Hennessy A, McLean M (2014). Cardiovascular disease in women: the significance of hypertension and gestational diabetes during pregnancy. Curr Opin Cardiol.

[27] Hashemi S, Ramezani Tehrani F, Mehrabi Y, Azizi F (2013). Hypertensive pregnancy disorders as a risk factor for future cardiovascular and metabolic disorders (Tehran Lipid and Glucose Study). J Obstet Gynaecol Res.

[28] Jarrell J, Chan S, Hauser R, Hu H (2005). Longitudinal assessment of PCBs and chlorinated pesticides in pregnant women from Western Canada. Environ Health.

[29] Takser L, Mergler D, Baldwin M, de Grosbois S, Smargiassi A, Lafond J (2005). Thyroid Hormones in Pregnancy in Relation to Environmental Exposure to Organochlorine Compounds and Mercury. Environ Health Perspect.

[30] Vo TT, Gladen BC, Cooper GS, Baird DD, Daniels JL, Gammon MD (2008). Dichlorodiphenyldichloroethane and polychlorinated biphenyls: intraindividual changes, correlations, and predictors in healthy women from the southeastern United States. Cancer Epidemiol Biomarkers Prev.

[31] Wittsiepe J, Schrey P, Lemm F, Eberwein G, Wilhelm M (2008). Polychlorinated dibenzo-p-dioxins/polychlorinated dibenzofurans (PCDD/Fs), polychlorinated biphenyls (PCBs), and organochlorine pesticides in human blood of pregnant women from Germany. J Toxicol Environ Health A.

[32] Ibarluzea J, Alvarez-Pedrerol M, Guxens M, Santa Marina L, Basterrechea M, Lertxundi A (2011). Sociodemographic, reproductive and dietary predictors of organochlorine compounds levels in pregnant women in Spain. Chemosphere.

[33] James RA, Hertz-Picciotto I, Willman E, Keller JA, Charles MJ (2002). Determinants of serum polychlorinated biphenyls and organochlorine pesticides measured in women from the child health and development study cohort, 1963–1967. Environ Health Perspect.

[34] Sarcinelli PN, Pereira ACS, Mesquita SA, Oliveira-Silva JJ, Meyer A, Menezes MA (2003). Dietary and reproductive determinants of plasma organochlorine levels in pregnant women in Rio de Janeiro. Environ Res.

[35] Nilsson E, Salonen Ros H, Cnattingius S, Lichtenstein P (2004). The importance of genetic and environmental effects for pre‐eclampsia and gestational hypertension: a family study. BJOG.

[36] Lin YP, Pessah IN, Puschner B (2013). Simultaneous determination of polybrominated diphenyl ethers and polychlorinated biphenyls by gas chromatography–tandem mass spectrometry in human serum and plasma. Talanta.

[37] Phillips DL, Pirkle JL, Burse VW, Bernert JT, Henderson LO, Needham LL (1989). Chlorinated hydrocarbon levels in human serum: effects of fasting and feeding. Arch Environ Contam Toxicol.

[38] Männistö T, Mendola P, Liu D, Leishear K, Sherman S, Laughon SK (2015). Acute air pollution exposure and blood pressure at delivery among women with and without hypertension. Am J Hypertens.

[39] Besis A, Samara C (2012). Polybrominated diphenyl ethers (PBDEs) in the indoor and outdoor environments: a review on occurrence and human exposure. Environ Pollut.

[40] Hooper K, McDonald TA (2000). The PBDEs: an emerging environmental challenge and another reason for breast-milk monitoring programs. Environ Health Perspect.

[41] Hofmeyr G, Belizan J, Dadelszen P (2014). Low‐dose calcium supplementation for preventing pre‐eclampsia: a systematic review and commentary. BJOG.

[42] Agrawal S, Fledderjohann J, Vellakkal S, Stuckler D (2015). Adequately diversified dietary intake and iron and folic acid supplementation during pregnancy is associated with reduced occurrence of symptoms suggestive of pre-eclampsia or eclampsia in Indian women. PLoS One.

[43] Brizzi P, Tonolo G, Esposito F, Puddu L, Dessole S, Maioli M (1999). Lipoprotein metabolism during normal pregnancy. Am J Obstet Gynecol.

[44] Letsky EA, de Swiet M (2008). Blood volume, haematinics, anaemia. Medical disorders in obstetrics practice.

[45] Ali AA, Rayis DA, Abdallah TM, Elbashir MI, Adam I (2011). Severe anaemia is associated with a higher risk for preeclampsia and poor perinatal outcomes in Kassala hospital, eastern Sudan. BMC Res Notes.

